# A comparative study of the interactions of cationic hetarenes with quadruplex-DNA forming oligonucleotide sequences of the insulin-linked polymorphic region (ILPR)

**DOI:** 10.3762/bjoc.10.314

**Published:** 2014-12-11

**Authors:** Darinka Dzubiel, Heiko Ihmels, Mohamed M A Mahmoud, Laura Thomas

**Affiliations:** 1 Department Chemie-Biologie, Universität Siegen, Adolf-Reichwein-Str. 2, 57068 Siegen, Germany

**Keywords:** DNA ligands, fluorescent probes, ILPR, nucleic acids, quadruplex DNA

## Abstract

The interactions of the ILPR sequence (ILPR = "insulin-linked polymorphic region") **a2** [d(ACAG_4_TGTG_4_ACAG_4_TGTG_4_)] with [2.2.2]heptamethinecyanine derivatives **1a–e** and with the already established quadruplex ligands coralyne (**2**), 3,3′-[2,6-pyridinediylbis(carbonylimino)]bis[1-methylquinolinium] (**3**), 4,4′,4′′,4′′′-(21*H*,23*H*-porphine-5,10,15,20-tetrayl)tetrakis[1-methylpyridinium] (**4**), naphtho[2,1-*b*:3,4-*b*′:6,5-*b*′′:7,8-*b*′′′]tetraquinolizinium (**5**) and thiazole orange (**6**) were studied. It is demonstrated with absorption, fluorescence and CD spectroscopy that all investigated ligands bind with relatively high affinity to the ILPR-quadruplex DNA **a2** (0.2–5.5 × 10^6^ M^−1^) and that in most cases the binding parameters of ligand-ILPR complexes are different from the ones observed with other native quadruplex-forming DNA sequences.

## Introduction

**Scheme 1:**
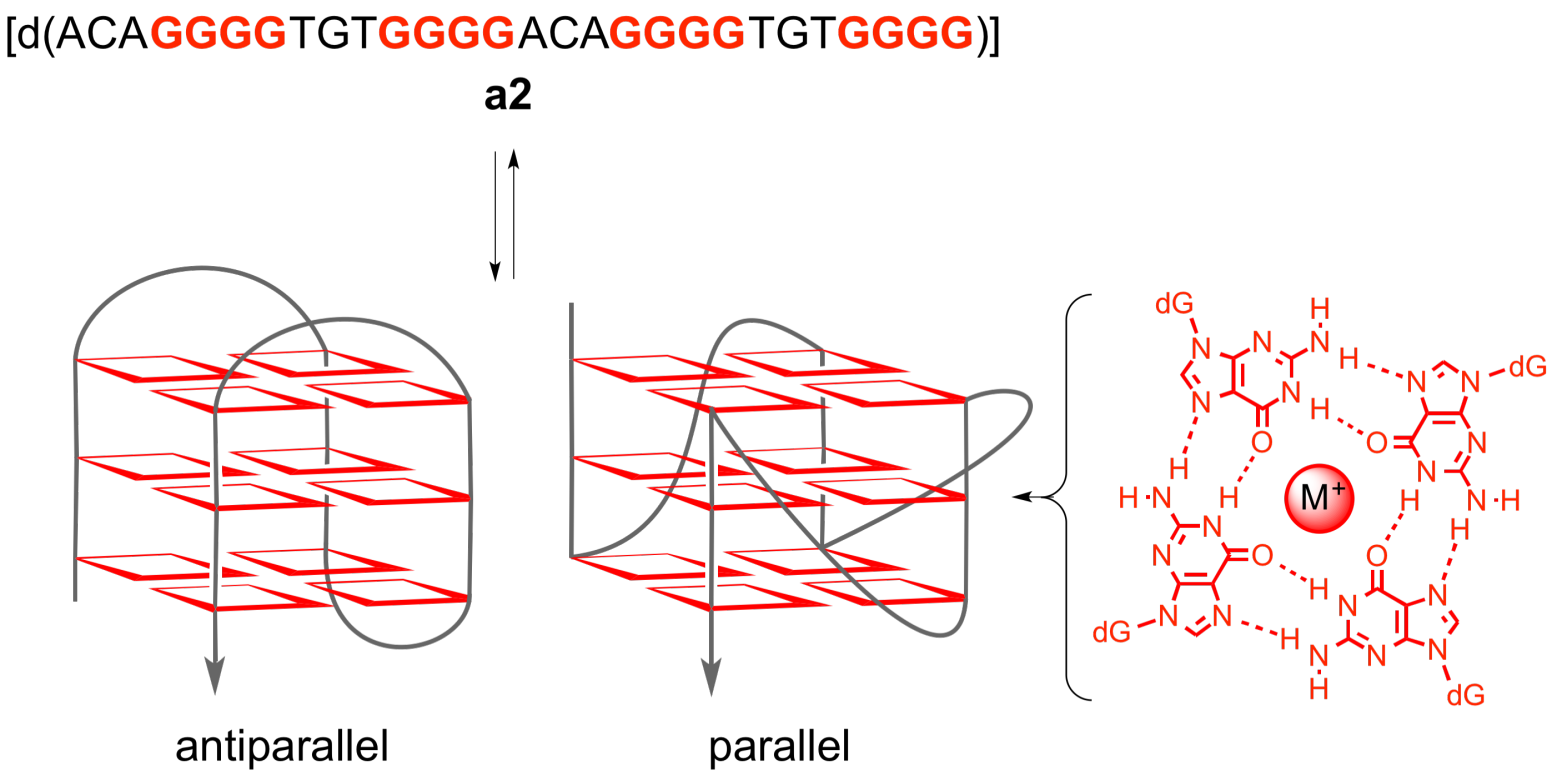
Equilibrium between single-stranded ILPR-DNA **a2** and its parallel and antiparallel quadruplex form.

The “insulin-linked polymorphic region” (ILPR) is a physiologically relevant G-rich DNA sequence that consists of repetitive DNA units with varying length and sequence [1]. The ILPR is located in the promoter region of the human insulin gene and is proposed to control the expression of the latter. It was shown that insulin-dependent diabetes mellitus (IDDM, type-I diabetes) is associated with the number of these minisatellites. Specifically, the number of these repeating units varies from ca. 160 in healthy humans to ca. 40 in IDDM patients [2]. From the 14 known repeating units of ILPR the variants **a** [d(ACAGGGGTGTGGGG)], **b** [d(ACAGGGGTCTGGGG)] and **c** [d(ACAGGGGTCCTGGGG)] occur most frequently; and **a** has the highest transcriptional activity [2–3]. It was shown that the sequences **a**–**c** form stable G-quadruplex structures in vitro and that this tendency is even enhanced by binding of the insulin protein [4–8]. In addition, it was demonstrated that the nucleotide sequence **a2**, [d(ACAG_4_TGTG_4_ACAG_4_TGTG_4_)], has parallel and antiparallel quadruplex forms that coexist under physiological conditions (Scheme 1) [9]. Notably, the biological relevance of ILPR quadruplexes has been suggested considering recent discoveries on the influence of quadruplex formation on the function of nucleic acids and the observation that insulin associates with quadruplex DNA [10]. In particular, it was assumed that the formation of quadruplex structures affects the transcriptional activity, because the position of the ILPR in the promoter region of the gene represents a stake close to the transcription process [9,11–12]. Furthermore, quadruplex structures may interfere with the replication process because of their thermal and mechanical stability. Namely, the force required to unfold the quadruplex is larger than the one to block helicases [9,13], such that quadruplex structures reduce the activity of these enzymes to greater extent than the corresponding duplex DNA [14]. There is also evidence that longer ILPR sequences may form multiple quadruplex structures that interact with each other, and it was speculated that the observed increase in replication errors in minisatellite regions is related with these higher-order DNA structures [15].

**Figure 1:**
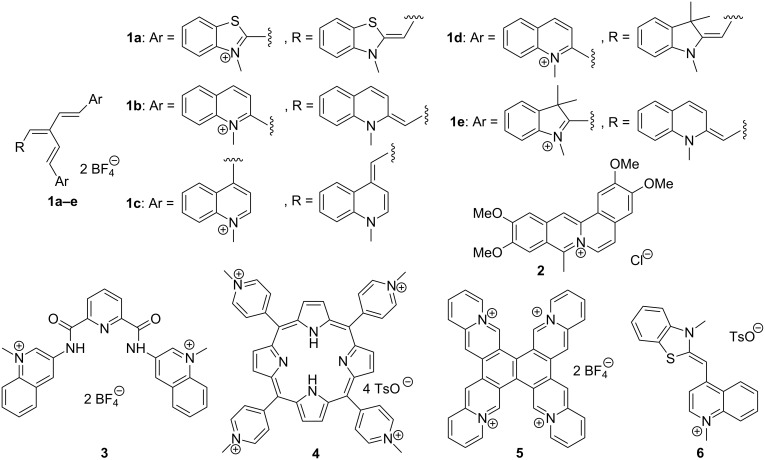
Structures of ligands **1**–**6** used in this study.

It has been demonstrated throughout the last decade that quadruplex DNA-binding ligands have a large potential to increase its stability towards unfolding and to influence the equilibrium between different quadruplex forms by stabilizing one particular quadruplex conformation [16–17]. Therefore, it is proposed that such ligands may interfere with physiological processes that involve quadruplex DNA. In this context, it is remarkable that systematic studies on the interaction of ligands with ILPR-DNA are rather rare. To the best of our knowledge there exists one study to develop a fluorescence screening method for the identification of selective quadruplex ligands in which, along with other sequences, an ILPR sequence has been used [18–19]. To fill this gap of knowledge, we investigated the interactions of a series of quadruplex ligands with the representative ILPR sequence **a2**. As we have demonstrated already that the [2.2.2]heptamethinecyanine dye binds selectively to quadruplex DNA and that its interaction with the quadruplex is indicated by a drastic emission light-up effect [20–21], we chose the [2.2.2]heptamethinecyanine derivatives **1a–e** [22] as ligands for this study. For a better comparison of data we also included the already established quadruplex ligands **2**–**6** [23–27] in this study (Figure 1).

## Results

### Thermal DNA-denaturation experiments

**Figure 2:**
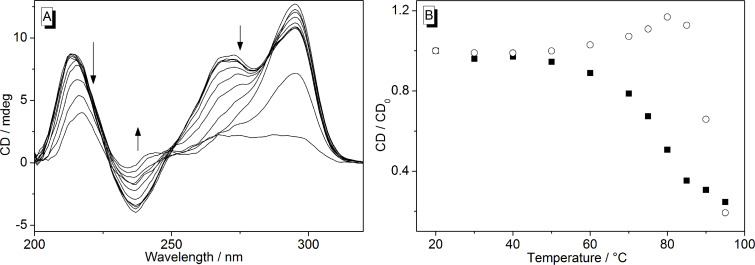
A) CD spectra of ILPR-DNA **a2** (20 μM) at different temperatures in potassium phosphate buffer (95 mM, pH 7.0). Arrows indicate the development of the bands with increasing temperature. B) Plot of the intensity changes (CD/CD_0_) of the CD signals at 265 nm (■) and 295 nm (○) versus temperature.

**Figure 3:**
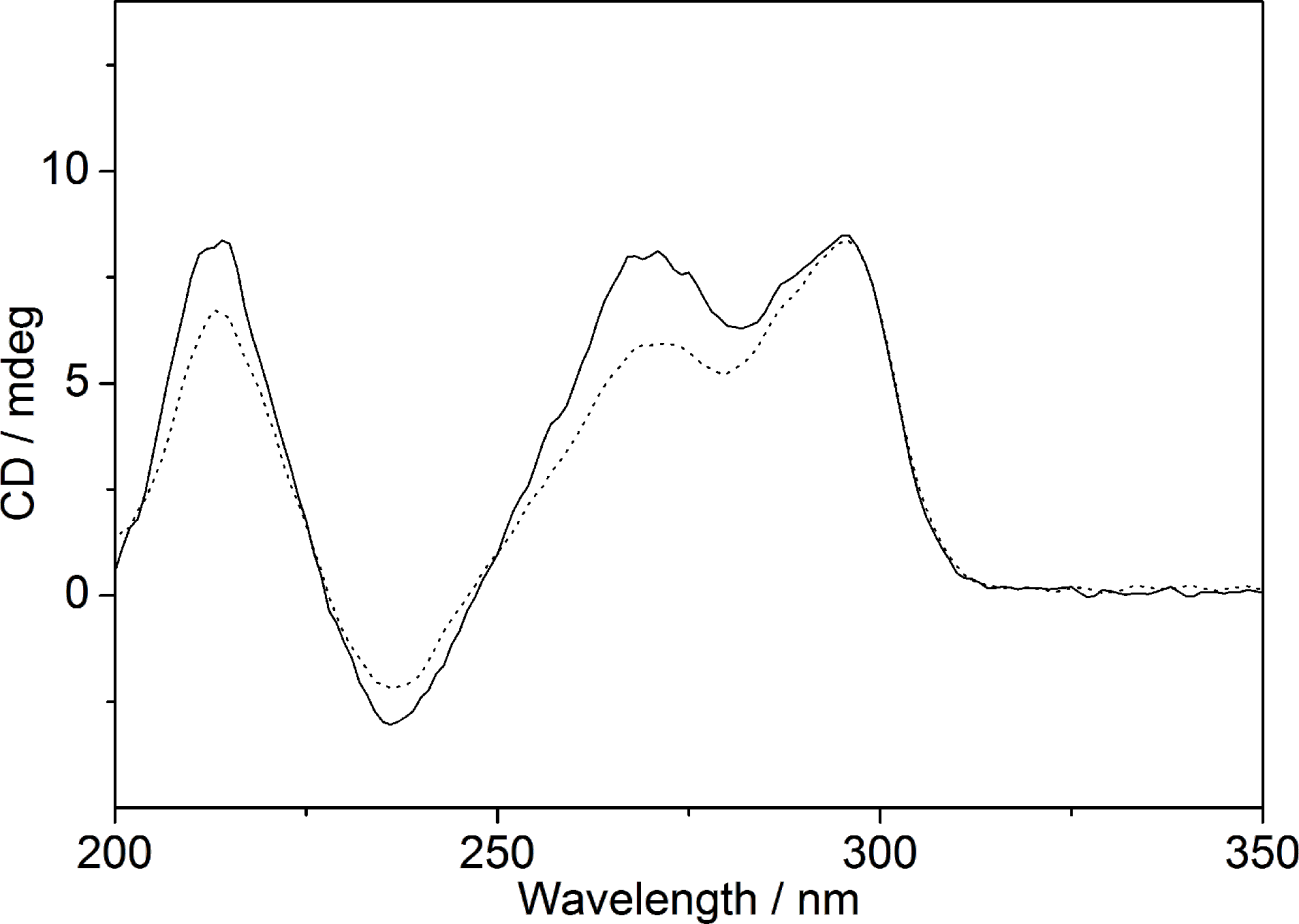
CD spectra of ILPR-sequence **a2** (20 µM) in potassium phosphate buffer (95 mM, pH 7.0; ^__^ : immediately after the preparation of the DNA stock solution; ···· : 2 d after the preparation).

The temperature-dependent structural changes of ILPR-DNA were analyzed by CD-spectroscopic and fluorimetric analysis. CD spectra were obtained from a solution of the ILPR quadruplex-forming oligonucleotide **a2** in K^+^-containing buffer (95 mM) in a temperature range from 20 to 95 °C. The oligonucleotide **a2** showed CD signals that are characteristic of a mixture of parallel (λ_max_ = 265 nm; λ_min_ = 235 nm) and antiparallel (λ_max_ = 295 nm) quadruplex structures (Figure 2A) [9,28]. With increasing temperature, the intensity of the maxima at 210 and 265 nm as well as the ones of the minimum at 235 nm decrease at *T* > 60 °C. On the other hand, the intensity of the signal at 295 nm slightly increases at *T* > 60 °C and then decreases at *T* > 80 °C (Figure 1A and 1B). These results are in agreement with literature data [9]; however, in the latter case the signal at 265 nm increased at 60 °C < *T* > 80 °C instead of the signal at 290 nm, presumably because different buffer solutions were used in the latter study*.* It should be noted that the CD spectrum of **a2** in potassium phosphate buffer (95 mM K^+^) changes slightly with time. Specifically, the intensity of the CD bands at 210, 235 and 265 nm decreases slowly within 48 h, whereas the maximum at 295 nm remains essentially the same in this time range (Figure 3).

**Figure 4:**
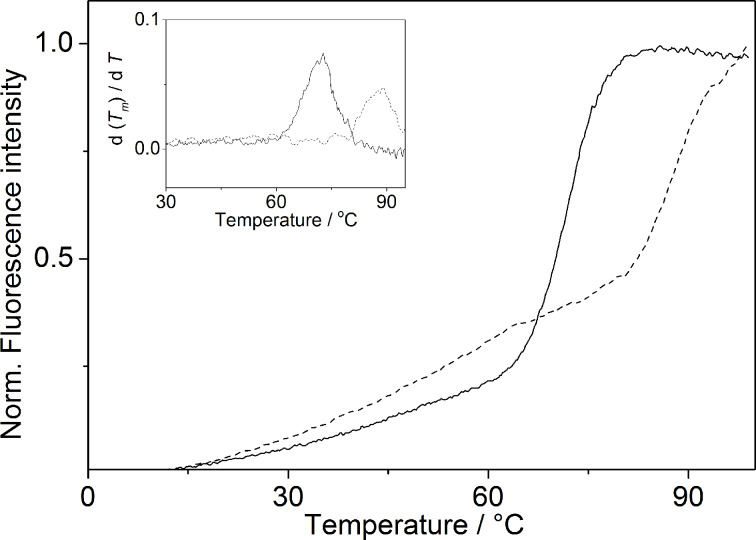
Fluorimetric monitoring of thermal DNA-denaturation of the ILPR quadruplex **Fa2T** (0.2 μM DNA concentration in oligonucleotide) in potassium phosphate buffer (····, 95 mM, pH 7.0) and KCl-LiCl-Na-cacodylate buffer (—, 10 mM K^+^, 90 mM Li^+^, pH 7.2). Inset: Plot of the first derivative *f'* (*T*) of the melting curves.

The thermal stability of the ILPR-DNA with respect to unfolding was also examined by emission spectroscopy in K^+^- and Na^+^-containing buffer, i.e., the melting temperature, *T*_m_, of the dye-labelled quadruplex-forming ILPR sequence **Fa2T** [fluorescein-d(ACAG_4_TGTG_4_ACAG_4_TGTG_4_)-tetramethylrhodamine] was determined by fluorimetric monitoring of the temperature-dependent Förster resonance energy transfer (FRET) between the dyes [29]. In sodium cacodylate buffer (10 mM Na^+^, 10 mM K^+^, 90 mM Li^+^) the melting curve of the DNA has a weak transition at 50 °C and a more pronounced one at *T*_m_ = 71.0 °C (Figure 4). The results of the melting experiments always refer to the latter transition. In potassium phosphate buffer (95 mM K^+^) the melting temperature of the DNA is *T*_m_ = 87.8 °C. This temperature is relatively high, so that further stabilization by a ligand leads to a *T_m_* close to the boiling point of the solvent. Therefore, studies of ligand–DNA complexes were not performed in this medium.

The influence of the ligands **1**–**6** on the melting temperature of ILPR-DNA was determined by fluorimetric monitoring of the DNA melting curves of **Fa2T** at different ligand–DNA ratios, LDR (Table 1, Figures S1 and S2 in Supporting Information File 1). Most of the tested ligands, i.e. **1a**, **1c**, **1d**, **2**, **4**, **5**, and **6**, induced only a moderate increase of the melting temperature of the ILPR-quadruplex (Δ*T*_m_ = ca. 2–6 °C). Nevertheless, the addition of the cyanine derivative **1b** or the bis-quinolinium derivative **3** induced a significantly larger shift of the quadruplex melting temperature (**1b**: Δ*T*_m_ = 14.5 °C; **3**: 14.9 °C; LDR = 5.0).

**Table 1:** Shifts of melting temperature, Δ*T*_m,_ of quadruplex-DNA **Fa2T** induced by ligands **1**–**6** as determined from fluorimetric thermal DNA denaturation studies.

LDR	Δ*T*_m_ [°C] ^a^		

	1.3	2.5	5.0
	
**1a**	1.0	1.9	4.9
**1b**	0.8	7.1	14.5
**1c**	0.1	1.1	3.5
**1d**	0.5	4.0	3.7
**1e**	1.3	2.2	3.4
**2**	0.7	1.7	3.1
**3**	3.9	6.4	14.9
**4**	1.4	2.1	6.3
**5**	0.1	1.5	5.8
**6**	0.7	0.8	1.8

^a^Δ*T*_m_ of **Fa2T**; *c*_DNA_ = 0.2 µM (in oligonucleotide); KCl-LiCl-Na-cacodylate buffer (10 mM K^+^, 90 mM Li^+^, pH 7.2); λ_ex_ = 470 nm; λ_em_ = 515 nm; estimated error: ±0.5 °C of the given data.

### Photometric and fluorimetric titrations

**Figure 5:**
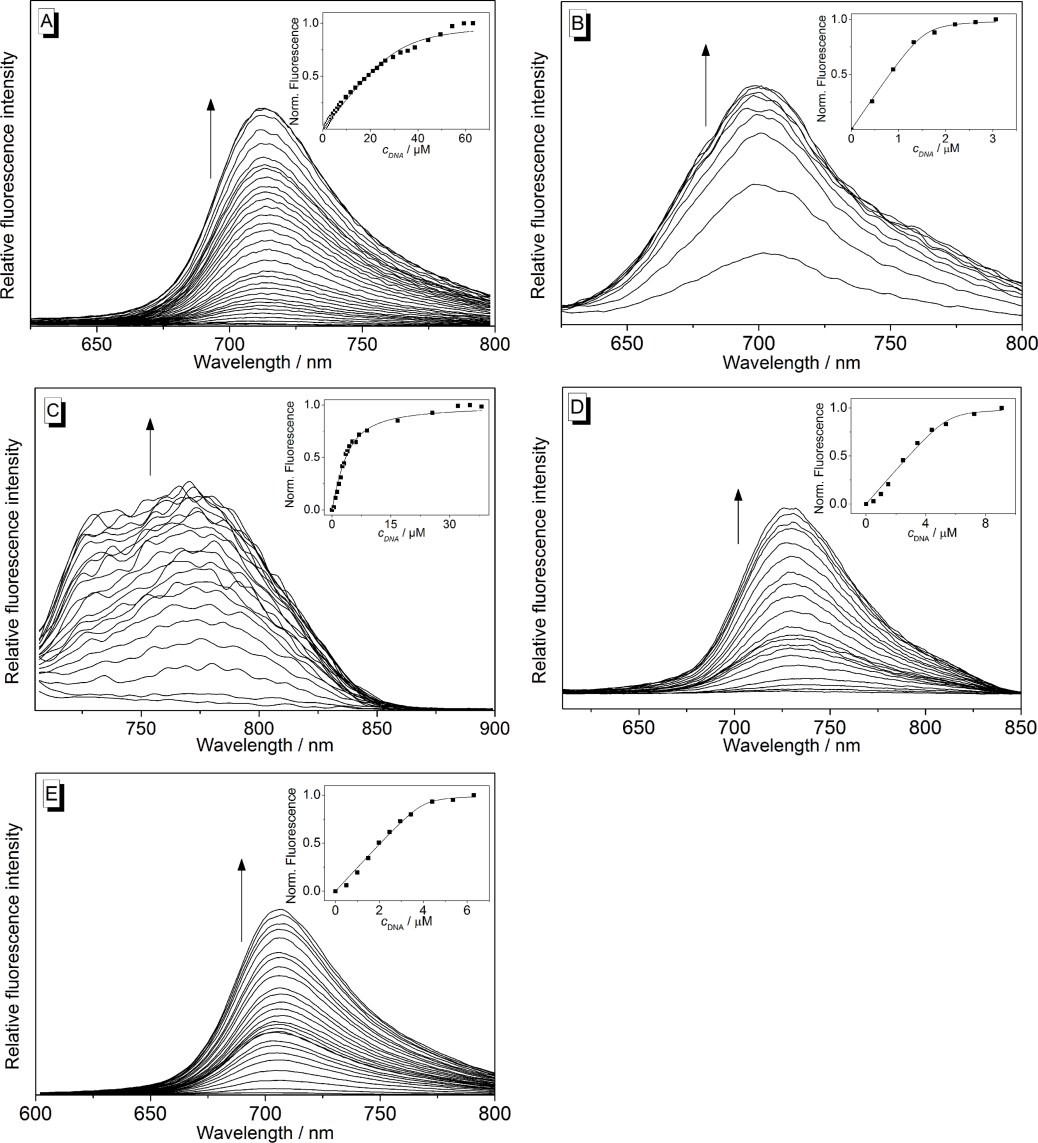
Fluorimetric titration of **1a** (A), **1b** (B), **1c** (C), **1d** (D) and **1e** (E) with **a2** in potassium phosphate buffer (95 mM, pH 7.0); A, B, E: *c*_Lig_ = 5.0 μM; C, D: *c*_Lig_ = 10 μM; A: λ_ex_ = 385 nm; B: λ_ex_ = 364 nm; C: λ_ex_ = 431 nm; D, E: λ_ex_ = 580 nm. Arrows indicate the development of the bands with increasing DNA concentration. Inset: Plot of the normalized emission intensity versus DNA concentration. Lines denote the best fit of experimental data to the theoretical model.

**Figure 6:**
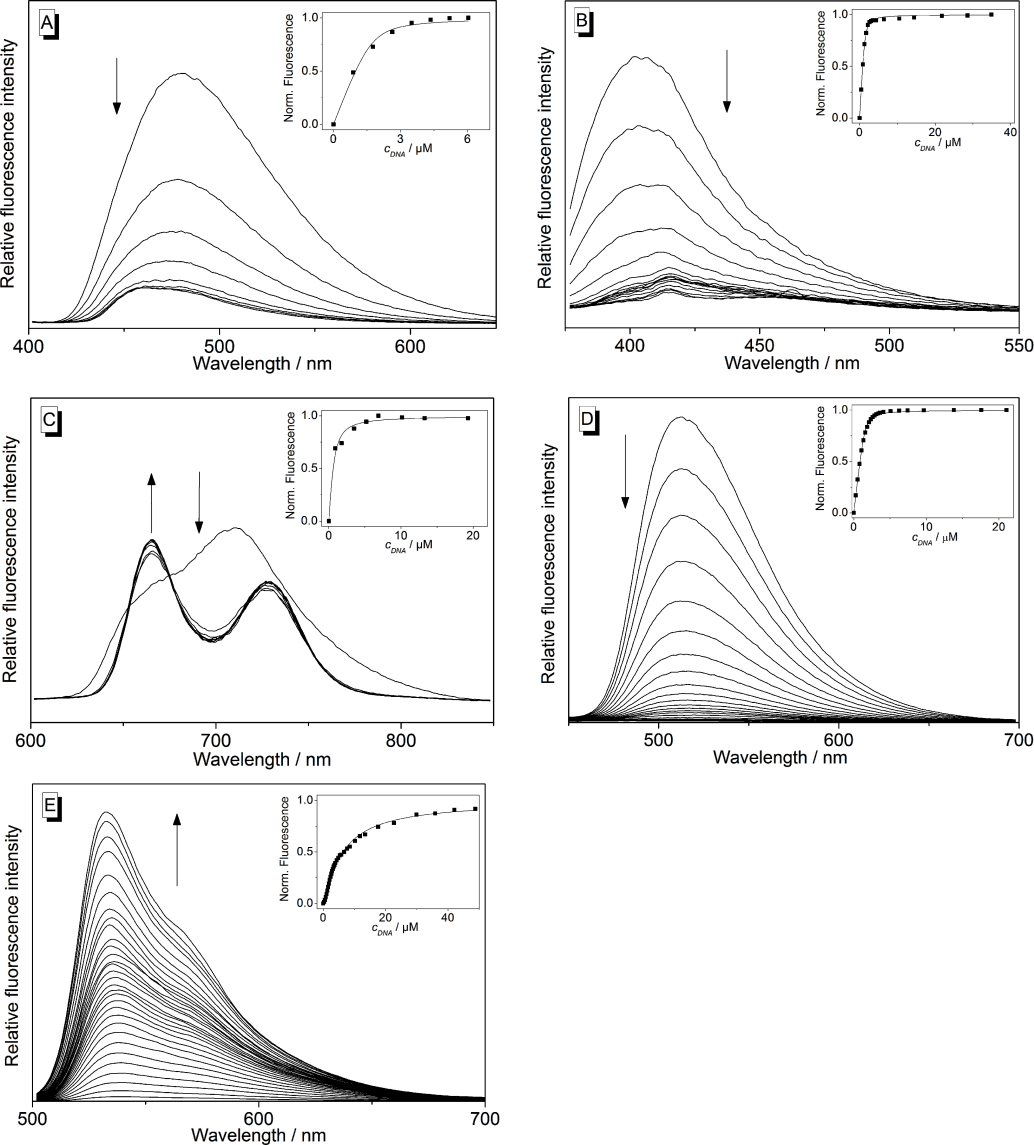
Fluorimetric titration of **2** (A), **3** (B), **4** (C), **5** (D) and **6** (E) with **a2** in potassium phosphate buffer (95 mM, pH 7.0); A, B, D, E: *c*_Lig_ = 5.0 μM; C: *c*_Lig_ = 10 μM; A, D: λ_ex_ = 385 nm; B: λ_ex_ = 364 nm; C: λ_ex_ = 431 nm; E: λ_ex_ = 490 nm. Inset: Plot of the change of the emission intensity versus DNA concentration. Lines denote the best fit of experimental data to the theoretical model. Arrows indicate the development of the bands with increasing DNA concentration.

The interactions of the ligands **1**–**6** with ILPR-DNA **a2** were further analyzed with photometric and fluorimetric titrations (Figure 5 and Figure 6, Table 2). In general, a hypochromic effect and a bathochromic shift of the absorption maximum of the ligand were observed upon addition of DNA (Supporting Information File 1, Figures S3 and S5). In the case of coralyne (**2**) and the tetraazoniahetarene derivative **5** this effect is only weakly pronounced. Only during the titration of the bis-quinolinium derivative **3** an isosbestic point developed at 364 nm.

**Table 2:** Absorption and emission properties of ligands **1**–**6** in the absence and presence of ILPR-DNA **a2**, and binding constants *K*_b_*.*

Ligand	λ_Abs_ [nm]^a^		λ_em_ [nm]^b^	*I*/*I*_0 _^c^	*K*_b_**^a2^** [10^6^ M^−1^]^d^	*K*_b_**^22AG^** [10^6^ M^−1^]^e^
		
	free	bound				

**1a**	624	635	712	92	1.2	0.8^f^
**1b**	577	648	700	17	2.7	0.2
**1c**	635	740	769	18	0.2	0.2
**1d**	626	648	718	128	5.5	0.9
**1e**	628	640	703	87	17	0.4
**2**	295	435	480	<0.1	2.5	n.d.^g^
**3**	346	374	404	<0.1	2.4	n.d.^g^
**4**	422	440	665, 728	n.d.^g^	2.2	2.9^h^
**5**	387	395	513	<0.1	3.5	1.8^h^
**6**	473	513	530	1766	0.3	2.1^i^

^a^Long-wavelength absorption maximum of the unbound and quadruplex-bound ligand. ^b^Emission maximum of bound ligand. ^c^Relative emission intensity, *I*/*I*_0_ (*I* = emission intensity of DNA-bound ligand at saturation, *I*_0_ = emission of unbound ligand). ^d^Binding constant of ligand–**a2** complex, *K*_b_**^a2^**, determined from fluorimetric titrations; DNA concentration in nucleotide bases. ^e^Binding constant of ligand–**22AG** complex, *K*_b_**^22AG^**, determined either from fluorimetric titrations or literature data; DNA concentration in oligonucleotide. ^f^Ref. [20]. ^g^n.d. = not determined. ^h^Ref. [23]. ^i^Ref [30].

The addition of ILPR-DNA **a2** to the cyanine derivatives **1a**–**e** and **6** resulted in a strong increase of the emission intensity (light-up effect) with increasing concentration of **a2** (Figure 5 and Figure 6E). This effect was most pronounced in the case of thiazole orange (**6**) whose emission intensity increases by a factor of *I*/*I*_0_ = 1766. Within the series of heptamethine cyanine dyes **1a**–**e**, the derivative **1d** exhibits the largest light-up factor with *I*/*I*_0_ = 128. In contrast, the emission of coralyne (**2**), bis-quinolinium derivative **3** and the tetraazoniahetarene derivative **5** was quenched upon addition of **a2** (Figure 6A, 6B, and 6D; Table 2). The data of the fluorimetric titrations was used to estimate the binding constants *K*_b_**^a2^** from a fit of the experimentally determined binding isotherms to the theoretical model [31] with resulting binding constants in the range of 0.2–5.5 × 10^6^ M^−1^ (Table 2). Notably, the development of the emission intensity of ligands **1d** and **1e** showed two distinctly different trends during the titration with **a2**, which indicates two different binding modes (Supporting Information File 1, Figure S4). Specifically, at LDR values <1.1 (**1d**) or <0.8 (**1e**) the emission intensity is significantly less quenched as compared to the beginning of the titration. The binding constants of the ligand–DNA complexes formed at the beginning of the titrations were estimated assuming that the emission intensities of **1d** und **1e** at LDR = 1.1 and 0.8 essentially represent the one of the bound ligand in this particular binding mode. Hence, the binding constants were determined from the respective binding isotherms (inset in Figure 5D and 5E; **1d**: *K*_b_**^a2^** = 5.5 × 10^6^ M^−1^; **1e**: *K*_b_**^a2^** = 1.7 × 10^7^ M^−1^). For comparison, fluorimetric titrations of the telomeric quadruplex **22AG** [5'-A(GGGTTA)_3_GGG-3'] to the cyanine derivatives **1b**–**e** were performed and the binding constants were obtained from the analysis of these data (Table 2; Figure S6 in Supporting Information File 1).

**Figure 7:**
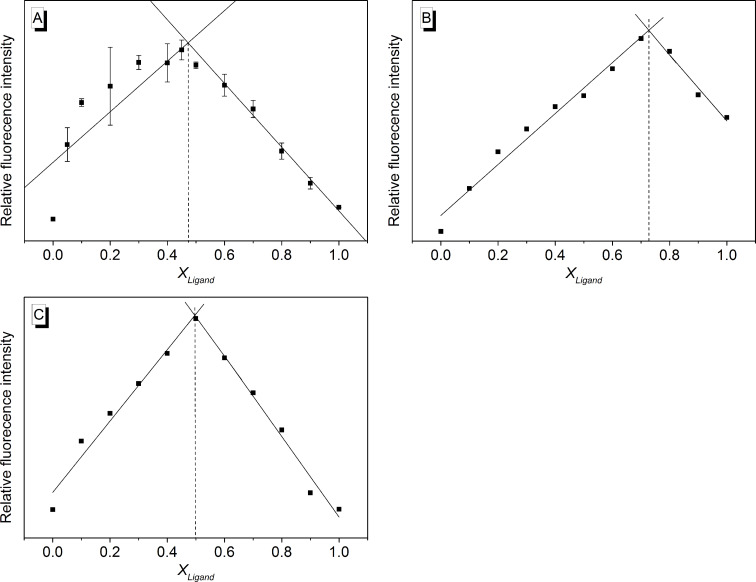
Job plot from fluorimetric analysis of mixtures of ligands **1e** (A), **4** (B) and **6** (C) with ILPR-DNA **a2** (*c*_Ligand_ + *c*_DNA_ = 10.0 μM) in potassium phosphate buffer (95 mM, pH 7.0); *X*_Ligand_ = mole fraction of the ligand; A: λ_ex_ = 580 nm; B: λ_ex_ = 431 nm; C: λ_ex_ = 490 nm; *T* = 20 °C.

The stoichiometry of the complexes of the ILPR-DNA **a2** with the representative ligands **1e**, **4** and **6** was determined with the continuous variations method (Job plot analysis). For that purpose, the emission intensity was plotted versus the mole fraction of the ligand, *X*_Ligand_*,* at constant total concentration of ligand and DNA (Figure 7). The graphically obtained maxima, i.e., the intercept of linearly fitted ascending and descending curve segments, were located at 0.48, 0.73 and 0.50 for ligands **1e**, **4** and **6**, respectively. Within the error margin these data correspond to a binding stoichiometry of 1:1 for complexes of **1e** and **6** with DNA **a2**, and a stoichiometry of 3:1 for the assembly of the porphyrin derivative **4** with the ILPR-DNA. However, it should be noted that strong fluctuations in the Job plot of the complex formation between ligand **1e** and ILPR-DNA **a2**, especially between *X*_Ligand_ = 0–0.4, indicate strong heterogeneous binding under these conditions.

### CD spectroscopic analysis of ligand–DNA interactions

**Figure 8:**
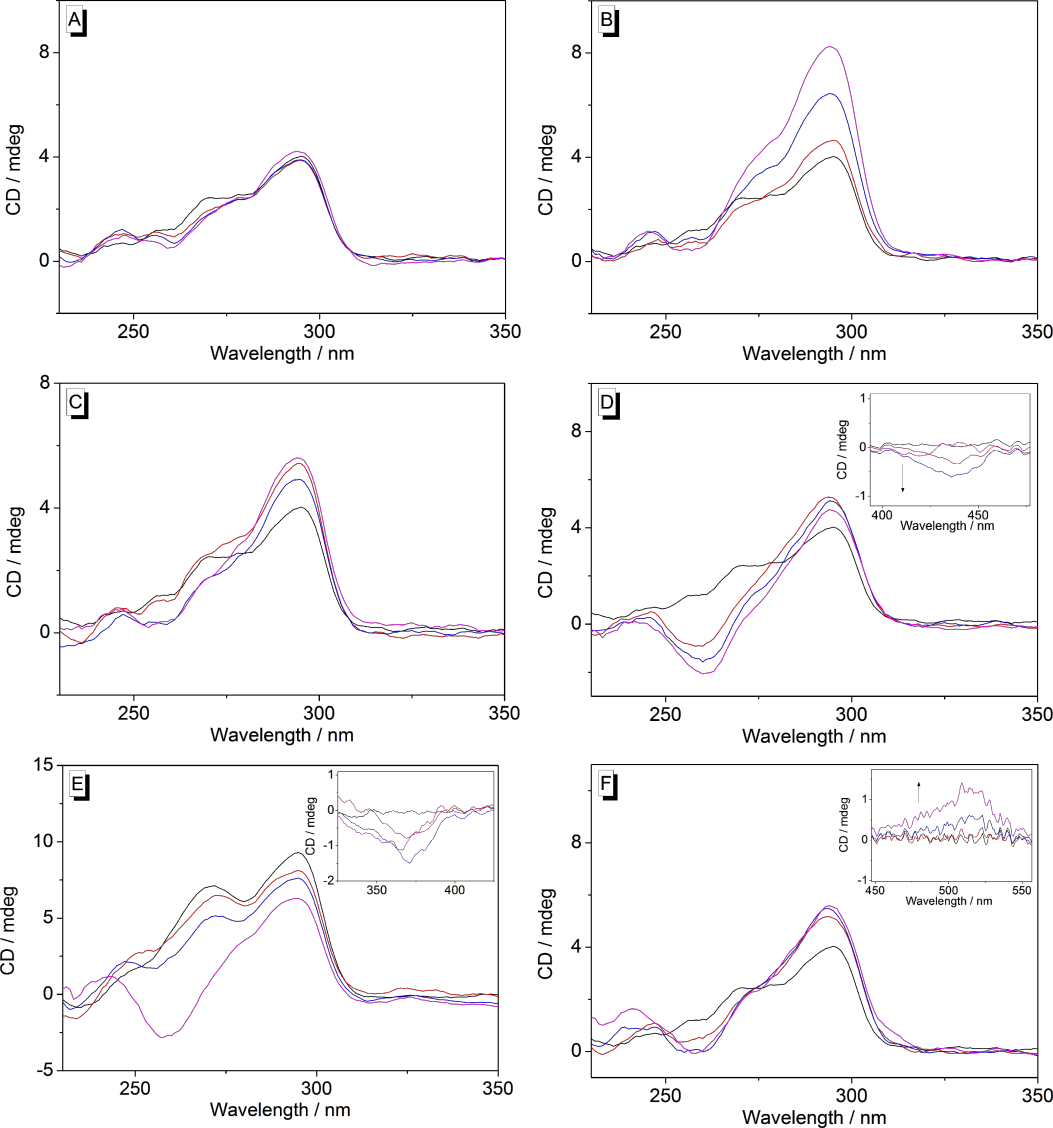
CD spectra of ILPR-DNA **a2** in the presence of **1d** (A), **1e** (B), **2** (C), **4** (D), **5** (E) and **6** (F) at LDR = 0 (black), 0.5 (red), 1 (blue), 2 (magenta); *c*_DNA_ = 20 μM in potassium phosphate buffer (95 mM, pH 7.0); *T =* 20 °C. Inset: Magnified ICD signals of the bound ligands (magnification factor: ca. 10).

The interactions of the ligands **1d**, **1e**, **2**, **4**–**6** with ILPR-DNA **a2** were analyzed by CD spectroscopy (Figure 8, Figure S7 in Supporting Information File 1). In most cases, the weak CD signal at 265 nm decreases and the CD signal at 295 nm increases slightly when the ligands were added. Upon addition of the cyanine derivative **1e** to ILPR-DNA, the intensity of the peak at 295 nm increased, whereas the peak at 265 nm slightly decreased. On the other hand, only very small, insignificant changes were observed on titration of **1d** to ILPR-DNA (Figure 8, Figure S7 in Supporting Information File 1). Notably, the addition of the porphyrin **4** and tetraazoniahetarene **5** to ILPR-DNA **a2** has a strong effect on the positive CD signal at 265 nm, leading to the development of a negative band at 260 nm at high LDR ratio (Figure 8D and 8E). In addition, induced CD (ICD) signals were observed only in the absorption range of compounds **4**–**6**.

## Discussion

Generally, it is possible to distinguish between different G-quadruplex DNA forms with the aid of CD spectroscopy [32]. Hence, in agreement with literature data [9–10], the characteristic bands at 235, 265 and 295 nm (Figure 2) show that the employed ILPR-DNA **a2** exists as a mixture of parallel (265 nm) and antiparallel (295 nm) G-quadruplex structures in potassium phosphate buffer solution. The presence of different quadruplex forms is further supported by the temperature-dependent CD spectra that denote the different melting temperatures of the parallel and antiparallel forms (Figure 3). Although it has been reported that the population of the parallel form increases with a low concentration of K^+^ ions [9]; in our hands it turned out to be dependent also on handling procedure. Hence, if the ILPR-quadruplex **a2** is heated to 97 °C and then cooled immediately to room temperature within 30 s, the CD spectrum of the solution shows a stronger CD signal at 265 nm than at 295 nm [9]. In contrast, if the ILPR-quadruplex **a2** is cooled slowly from 97 °C to room temperature with a rate of 0.5 °C/min the signal at 295 nm is higher than the one at 265 nm [10]. These results clearly demonstrate a delicate equilibrium between the different quadruplex forms and some kinetic barriers that result in rather slow interconversion processes. Nevertheless, to provide comparable data, in this study we used slowly cooled ILPR-quadruplex **a2** to ensure that we have the same relative population of parallel and antiparallel forms in each experiment.

The fluorimetric thermal DNA denaturation experiments show that the ligands **1b** and **3** induce the highest stabilization of ILPR-quadruplex under the employed conditions (Δ*T*_m_ = 14.5 °C, 14.9 °C respectively, Table 1), whereas the ligands **1a**, **1c**–**e**, **2**, **4** and **5** stabilize this DNA just moderately (Δ*T*_m_ = ca. 3–6 °C). Although the cyanine derivatives **1d** and **1e** show the highest binding affinity towards ILPR-quadruplex, these ligands induce only a moderate shift of its melting temperature (Table 2). This apparent contradiction may be the result of different buffer solutions used in different types of experiments, because the buffer composition, especially the K^+^ concentration, affects the population of parallel and antiparallel forms [9]. In the thermal denaturation experiment, the population of the parallel forms increases, because a low K^+^ concentration buffer was used (vide supra). On the other hand, a larger concentration of K^+^ was used during the fluorimetric titration leading to a higher fraction of the antiparallel form [9–10]. Apart from the derivatives **1c** and **6** the binding constants of the cyanine dyes with the ILPR-DNA are larger than those observed with the telomeric quadruplex **22AG** (Table 2). For example, the cyanine derivative **1e** shows the most significant difference between the binding affinity towards ILPR and the telomeric quadruplex (**a2**: *K*_b_**^a2^** = 1.7 × 10^7^ M^−1^; **22AG**: *K*_b_**^22AG^** = 3.9 × 10^5^ M^−1^). Although thiazole orange (**6**) has a relatively high binding constant with telomeric quadruplex DNA, it has a lower affinity towards the ILPR quadruplex under the same conditions. In addition, thiazole orange (**6**) shows the least pronounced stabilization effect in the thermal denaturation experiment (Table 2). As cyanine dyes bind to quadruplex DNA by terminal π-stacking [20], which is in agreement with the observed binding stoichiometry of 1:1 (Figure 7), the different composition of the loops in ILPR-quadruplex (ACA and TGT in ILPR versus TTA in telomeric quadruplex) may affect the binding affinity of ligands towards the ILPR-DNA. On the other hand, the porphyrin **4** and the tetraazoniahetarene derivative **5** show similar binding affinities towards the ILPR-quadruplex thus resembling their interactions with the telomeric **22AG** quadruplex [23]. This observation indicates that these ligands may bind in similar types of binding sites. It should be noted that in the case of the porphyrin **4** the ligand–**a2** stoichiometry of 1:3 (Figure 7) indicates additional stacking of the ligand along the phosphate backbone of the ILPR-DNA.

The cyanine derivatives **1a**, **1d**, **1e** and **6** show significant light-up effects upon association with the ILPR quadruplex (Table 2). Although thiazole orange (**6**) shows the highest light-up factor (*I*/*I*_0_ = 1766), it has a low selectivity over different types of DNA [33]. Thus, the use of thiazole orange for the selective fluorimetric detection of quadruplex DNA is rather limited. On the other hand, the cyanine derivatives **1a**, **1d** and **1e** show high selectivity towards quadruplex as compared to double stranded DNA [20]. Therefore, these ligands may be used as probes for the detection of quadruplex DNA.

Although the CD-spectroscopic analysis only provides qualitative information about the binding between the ligand and the DNA, they may be used to identify some general trends. Thus, the CD-spectroscopic analysis revealed that some of the ligands induce a very small (**1d**, **6**), moderate (**2**), or even strong (**1e**) increase of the CD band of the DNA **a2** at 295 nm. As this particular band has been assigned to the antiparallel quadruplex structure of **a2** [9–10] the induced increase may indicate a stabilization of this DNA structure upon complex formation. Moreover, a comparison with the telomeric quadruplex **22AG** shows that the ligands **1d**, **1e** and **6** induce the same effect on the CD band of this quadruplex at 295 nm (Supporting Information File 1, Figure S8). At the same time, the ICD of thiazole orange (**6**) in the presence of **22AG** is stronger than that with **a2** (Figure 8F and Figure S8C, Supporting Information File 1) indicating a stronger interaction of the ligand towards the telomeric quadruplex. This observation is in agreement with the higher affinity of **6** towards **22AG** (Table 2). Notably, coralyne (**2**) has a significantly different influence on the CD spectrum of **22AG** as compared to **a2.** In contrast to ILPR-DNA **a2**, the band of **22AG** at 295 nm decreases upon addition of compound **2**, along with a strong ICD signal of the ligand [34]; thus indicating a different binding mode of coralyne (**2**) with **a2** and **22AG**.

Notably, the CD-spectroscopic analysis shows a marginal effect of **1d** and **1e** on the ILPR-DNA **a2**. Nevertheless, as the other experimental data confirm the association of this ligand to the quadruplex it may be concluded that the complex formation between **a2** and **1d** and **1e** does not lead to a significant change of the DNA structure.

In the case of ligands **2**, **4**, **5** and **6**, a negative CD band developed at 265 nm together with a weak positive band with a maximum at ca. 240 nm upon association with quadruplex **a2** (Figure 8). Along with the increase of the CD signal at 295 nm these developments may indicate the pronounced formation of an antiparallel quadruplex form [35]. Furthermore, the CD spectrum of **a2** in the presence of the ligands **2**, **4**, **5** and **6** displays roughly the same maxima as the antiparallel quadruplex with only two stacked G-quartet planes that is formed by the thrombin binding aptamer (TBA) [d(G_2_T_2_G_2_TGTG_2_T_2_G_2_)], though with varying intensities and slightly different shifts [32]. In addition, it has been suggested that the porphyrin **4** also binds to an antiparallel quadruplex form that consists of just two stacked G-quartets and more extended loop structures [36], the so-called Form 3. Presumably, the sterically demanding ligand **4** fits well into the larger binding sites of the Form 3 quadruplex [37–38]. Considering all these observations it may be very carefully proposed that the ILPR-DNA **a2** also forms structures with only two stacked G-quartets that are stabilized upon association of the ligands **2**, **4**, **5** and **6**. Nevertheless, this proposed folding pattern of the oligonucleotide **a2** needs to be confirmed in a more detailed structure analysis by high-resolution NMR spectroscopy.

## Conclusion

This study represents the first comparative investigation of the interaction of different quadruplex ligands with an oligonucleotide sequence from the ILPR. In summary, it is demonstrated that in most cases the binding parameters of ligand-ILPR complexes are different from the ones observed with other native quadruplex-forming DNA sequences. All ligands under investigation show a high binding affinity towards the ILPR quadruplex. Cyanine derivatives – except thiazole orange (**6**) – have a higher affinity towards ILPR quadruplex than towards the telomeric quadruplex. From these results it may be concluded that, in principle, the association of exogenous ligands with the ILPR-DNA may assist or interfere with the biological activity of this physiologically relevant DNA sequence.

## Experimental

### Materials

Coralyne (**2**), porphyrin **4** and thiazole orange (**6**) were commercially obtained and used without further purification (**2**: Acros Organics, Geel, Belgium; **4**, **6**: Fluka Chemie AG, Buchs, Switzerland). The [2.2.2]heptamethine cyanine dyes **1a**–**e** [22], bis-quinolinium **3** [39] and tetraazoniahetarene **5** [23] were synthesized according to published procedures. Oligodeoxyribonucleotides (HPLC purified) **a2** [d(ACAG_4_TGTG_4_ACAG_4_TGTG_4_)] and **Fa_2_T** [d(Fluo-ACAG_4_TGTG_4_ACAG_4_TGTG_4_-Tamra)] were purchased from Metabion Int. AG (Planegg/Martinsried). The ILPR-DNA **a2** or **Fa_2_T** were dissolved in the proper buffer, heated to 95 °C for 5 min, cooled slowly over 4 hours to room temperature and used two days after storing. Potassium phosphate buffer: 25 mM K_2_HPO_4_, 70 mM KCl; adjusted with 25 mM KH_2_PO_4_ to pH 7.0; sodium cacodylate buffer: 10 mM Na(CH_3_)_2_AsO_2_·3H_2_O, 10 mM KCl, 90 mM LiCl; pH 7.2–7.3.

### Equipment

Absorption spectroscopy: Varian Cary 100 Bio spectrophotometer; emission spectroscopy: Varian Cary Eclipse; CD spectroscopy: Chirascan CD-spectrometer, Applied Photophysics.

### Methods

All photometric and fluorimetric titrations were performed in thermostated quartz cuvettes at 20 °C. Titrant solutions were freshly prepared by dilution from the stock solution. Spectrophotometer slit widths were 2 nm for photometric experiments and 5 nm for fluorimetric experiments. In fluorimetric experiments, the spectra were smoothed with implemented moving-average function by a factor of 5. The binding constants were determined by fitting the binding isotherms from the fluorimetric titrations to the established theoretical model according to the independent-site model (Equation 1) [31].

[1]
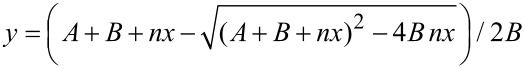


Where *y* is the normalized intensity; *A* = 1/*K*_b_; *B* = *c*_Lig_, and *n* is the number of the binding sites per quadruplex DNA.

Thermal denaturation experiments were performed according to the published procedure [20].

For Job plot analysis (continuous variations method), a series of samples was prepared with a constant sum of concentrations at 10.0 μM, but with varying concentrations of ligand and ILPR quadruplex. The fluorescence spectra were recorded for each sample with λ_ex_ = 580 nm for cyanine derivative **1e**, and λ_ex_ = 431 nm for porphyrin derivative **4** and λ_ex_ = 490 nm for thiazole orange (**6**). The maximum fluorescence intensity was plotted versus the molar fraction of the corresponding ligand, *X*_Ligand_. For the determination of the maximum the ascending and descending segments of the curve were fitted to linear lines, respectively, and the intercept of both lines denotes the maximum and thus the stoichiometry of the complex.

For the CD experiments five samples were prepared with fixed ILPR-DNA concentration (*c*_DNA_ = 20.0 μM). In four of the samples different amounts of ligand were added to obtain different ligand concentrations (5, 10, 20, 40 μM). CD signals were recorded with a band width of 1 nm, a recording speed of 1 nm s^−1^ and a time per data point of 0.5 s. For the temperature dependent circular dichroism (CD) experiments, solutions of the ILPR-quadruplex **a2** (20 μM) and the ligand (20 μM) were prepared in potassium phosphate buffer (95 mM, pH 7.0) and measured after an equilibration time of 24 h. After each measurement, the temperature was increased by 5 °C or 10 °C, after the temperature has been kept to an equilibration time of 10 min.

## Supporting Information

Figures of fluorimetric DNA denaturation experiments, photometric titration of **a2** ILPR-DNA into ligands **1**–**6**, photometric and fluorimetric titration of **22AG** into ligands **1b**–**e**, plots of the CD intensity change of **a2** ILPR-DNA in presence of ligands **1**–**6** and CD spectra of **22AG** in presence of ligands **1d**, **1e** and **6** are provided.

File 1 Additional experimental data.

## References

[1] Bell G I, Horita S, Karam J H (1984). Diabetes.

[2] Kennedy G C, German M S, Rutter W J (1995). Nat Genet.

[3] Rotwein P, Yokoyama S, Didier D K, Chirgwin J M (1986). Am J Hum Genet.

[4] Catasti P, Chen X, Moyzis R K, Bradbury E M, Gupta G (1996). J Mol Biol.

[5] Lew A, Rutter W J, Kennedy G C (2000). Proc Natl Acad Sci U S A.

[6] Schonhoft J D, Das A, Achamyeleh F, Samdani S, Sewell A, Mao H, Basu S (2010). Biopolymers.

[7] Gerasimov J Y, Schaefer C S, Yang W, Grout R L, Lai R Y (2013). Biosens Bioelectron.

[8] Xiao J, Carter J A, Frederick K A, McGown L B (2009). J Sep Sci.

[9] Yu Z, Schonhoft J D, Dhakal S, Bajracharya R, Hegde R, Basu S, Mao H (2009). J Am Chem Soc.

[10] Timmer C M, Michmerhuizen N L, Witte A B, Van Winkle M, Zhou D, Sinniah K (2014). J Phys Chem B.

[11] De Messieres M, Chang J-C, Brawn-Cinani B, La Porta A (2012). Phys Rev Lett.

[12] Hammond-Kosack M C U, Dobrinski B, Lurz R, Docherty K, Kilpatrick M W (1992). Nucleic Acids Res.

[13] Perkins T T, Li H-W, Dalal R V, Gelles J, Block S M (2004). Biophys J.

[14] Liu J-q, Chen C-y, Xue Y, Hao Y-h, Tan Z (2010). J Am Chem Soc.

[15] Schonhoft J D, Bajracharya R, Dhakal S, Yu Z, Mao H, Basu S (2009). Nucleic Acids Res.

[16] Oganesian L, Bryan T M (2007). BioEssays.

[17] Murat P, Singh Y, Defrancq E (2011). Chem Soc Rev.

[18] Paramasivan S, Bolton P H (2008). Nucleic Acids Res.

[19] Yan J-W, Chen S-B, Liu H-Y, Ye W-J, Ou T-M, Tan J-H, Li D, Gu L-Q, Huang Z-S (2014). Chem Commun.

[20] Ihmels H, Thomas L (2013). Org Biomol Chem.

[21] Nanjunda R, Owens E A, Mickelson L, Alyabyev S, Kilpatrick N, Wang S, Henary M, Wilson W D (2012). Bioorg Med Chem.

[22] Reichardt C, Mormann W (1972). Chem Ber.

[23] Jäger K, Bats J W, Ihmels H, Granzhan A, Uebach S, Patrick B O (2012). Chem – Eur J.

[24] Pennarun G, Granotier C, Gauthier L R, Gomez D, Hoffschir F, Mandine E, Riou J-F, Mergny J-L, Mailliet P, Boussin F D (2005). Oncogene.

[25] Granzhan A, Ihmels H, Jäger K (2009). Chem Commun.

[26] Wang P, Leung C-H, Ma D-L, Yan S-C, Che C-M (2010). Chem – Eur J.

[27] Monchaud D, Allain C, Teulade-Fichou M-P (2006). Bioorg Med Chem Lett.

[28] Williamson J R (1994). Annu Rev Biophys Biomol Struct.

[29] Renčiuk D, Zhou J, Beaurepaire L, Guédin A, Bourdoncle A, Mergny J-L (2012). Methods.

[30] Monchaud D, Allain C, Teulade-Fichou M-P (2007). Nucleosides, Nucleotides Nucleic Acids.

[31] Stootman F H, Fisher D M, Rodger A, Aldrich-Wright J R (2006). Analyst.

[32] Vorlíčková M, Kejnovská I, Sagi J, Renčiuk D, Bednářová K, Motlová J, Kypr J (2012). Methods.

[33] Allain C, Monchaud D, Teulade-Fichou M-P (2006). J Am Chem Soc.

[34] Bhadra K, Kumar G S (2011). Biochim Biophys Acta.

[35] Paramasivan S, Rujan I, Bolton P H (2007). Methods.

[36] Martino L, Pagano B, Fotticchia I, Neidle S, Giancola C (2009). J Phys Chem B.

[37] Lim K W, Amrane S, Bouaziz S, Xu W, Mu Y, Patel D J, Luu K N, Phan A T (2009). J Am Chem Soc.

[38] Zhang H-J, Wang X-F, Wang P, Ai X-C, Zhang J-P (2008). Photochem Photobiol Sci.

[39] Dorazco-González A, Höpfl H, Medrano F, Yatsimirsky A K (2010). J Org Chem.

