# Supplementary material for: A comparative study of the interactions of cationic hetarenes with quadruplex-DNA forming oligonucleotide sequences of the insulin-linked polymorphic region (ILPR)
Source: Beilstein J Org Chem. 2014 Dec 11;10:2963–74. doi: 10.3762/bjoc.10.314 (PMC4273293; doi:10.3762/bjoc.10.314)
Supplement: File 1 — Additional experimental data. [file Beilstein_J_Org_Chem-10-2963-s001.pdf]

Supporting Information

for

A comparative study of the interactions of cationic hetarenes with quadruplex-DNA forming oligonucleotide sequences of the insulin-linked polymorphic region (ILPR)

Darinka Dzubiel, Heiko Ihmels*, Mohamed M. A. Mahmoud and Laura Thomas

Address: Department Chemie-Biologie, Universität Siegen, Adolf-Reichwein-Str. 2, 57068 Siegen, Germany

Author names in alphabetical order that does not reflect the specific contribution of each author.

Email: Heiko Ihmels - ihmels@chemie.uni-siegen.de

*Corresponding author

Additional experimental data

Table of content

Figure S1: Fluorimetric DNA denaturation experiments……………………………………………S3

Figure S2: Fluorimetric DNA denaturation experiments……………………………………………S4

Figure S3: Photometric titration………………………………………………………………………… S5

Figure S4: Plot of the change of the emission intensity……………………………………………….S6

Figure S5: Photometric titration……………………………………………………………………..… S7

Figure S6: Fluorimetric titration…………………………………………………………………..…… S8

Figure S7: Plot of the intensity change (CD/CD_o)……………………………………………………...S9

Figure S8: CD spectra of 22AG with different ligands ……………………………………………S10

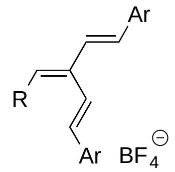
Ar
R
Ar BF4

1a–e

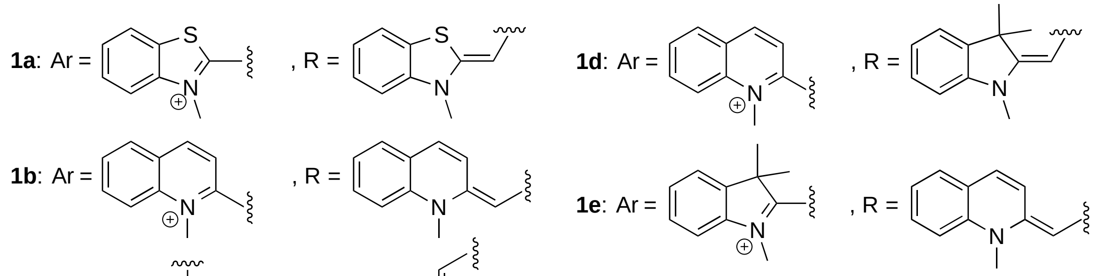
1a: Ar =
, R =
1b: Ar =
, R =
1d: Ar =
, R =
1e: Ar =
, R =

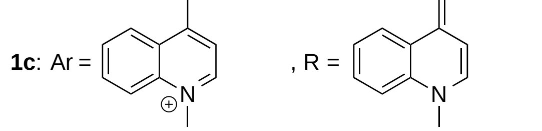
1c: Ar =
, R =

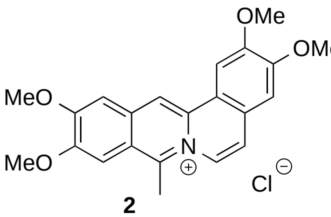
OMe
OMe
MeO
MeO
Cl

2

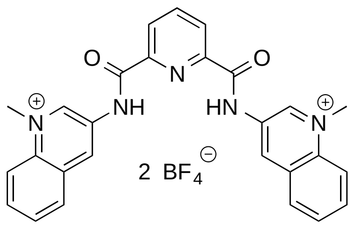
2 BF4

3

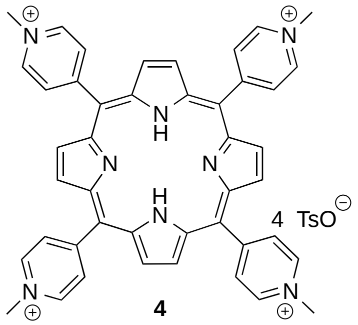
4 TsO

4

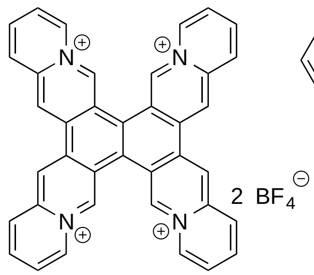
2 BF4

5

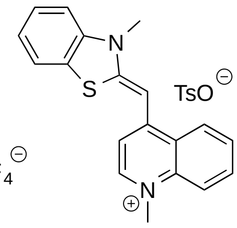
TsO

6

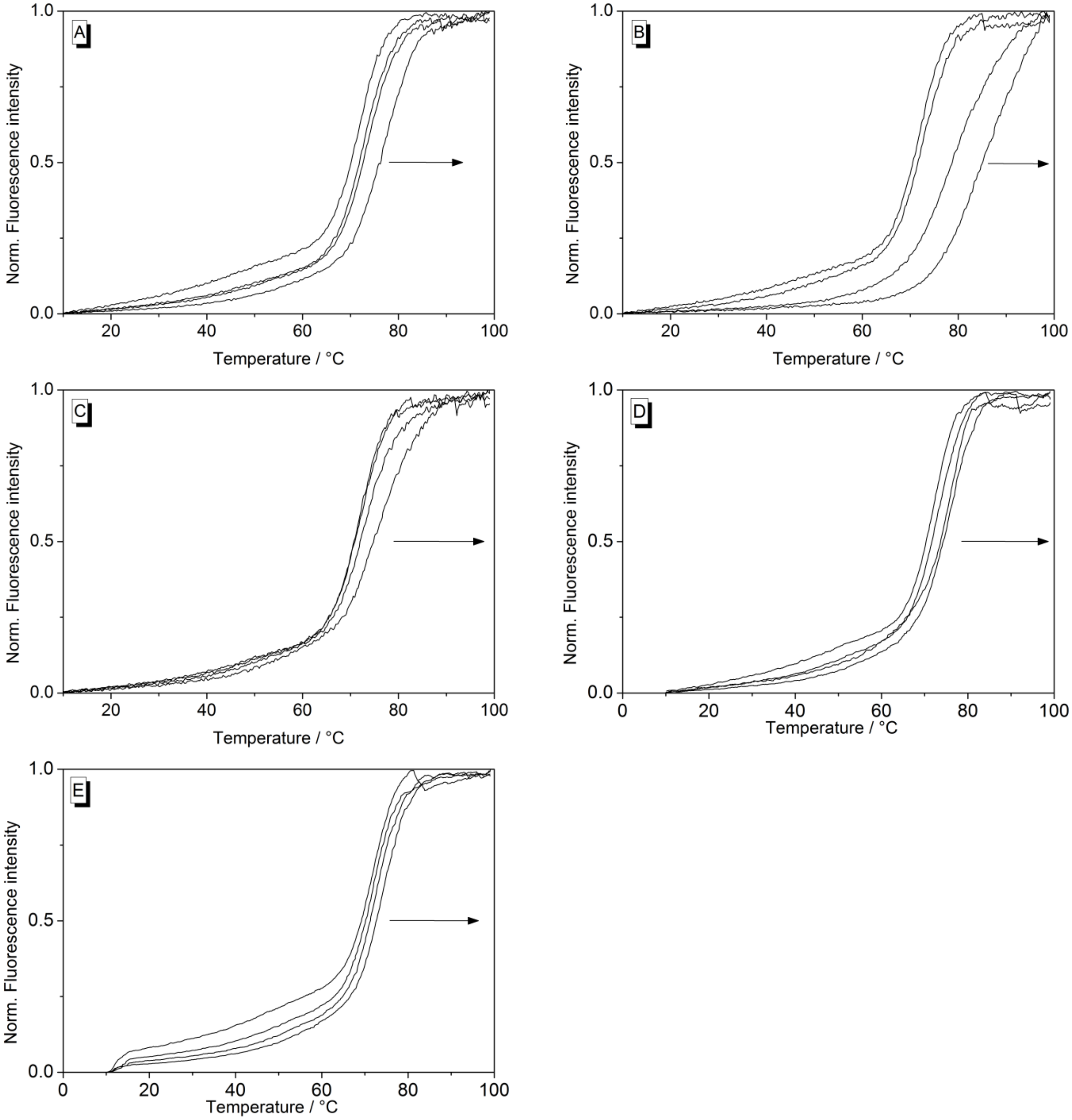

Figure S1: Fluorimetric DNA denaturation experiments of G-quadruplex **Fa_2T** (0.2 µM) in the presence of ligands **1a** (A), **1b** (B), **1c** (C), **1d** (D) and **1e** (E); *LDR*: 0, 1.25, 2.5, 5 (molar equivalents); KCl-LiCl-Na-cacodylate buffer (10 mM K^+, pH 7.2); λ_{ex} = 470 nm. The arrows indicate the evolution of the melting curve with increasing ligand concentration.

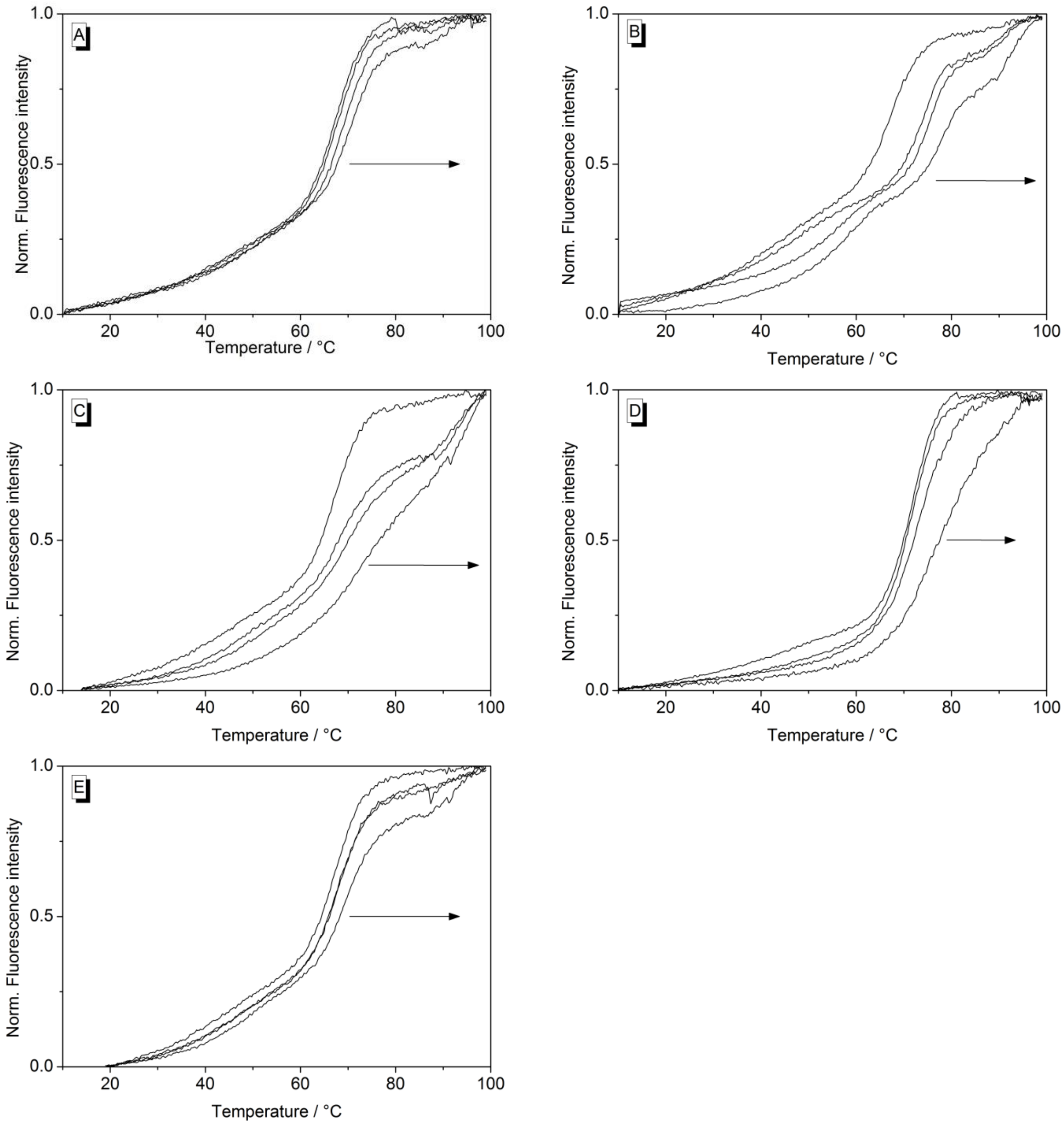

Figure S2: Fluorimetric DNA denaturation experiments of G-quadruplex **Fa$_2$T** (0.2 µM) in the presence of ligands **2** (A), **3** (B), **4** (C), **5** (D) and **6** (E); *LDR*: 0, 1.25, 2.5, 5 (molar equivalents); KCl-LiCl-Na-cacodylate buffer (10 mM K^+, pH 7.2); λ_{ex} = 470 nm. The arrows indicate the evolution of the melting curve with increasing ligand concentration.

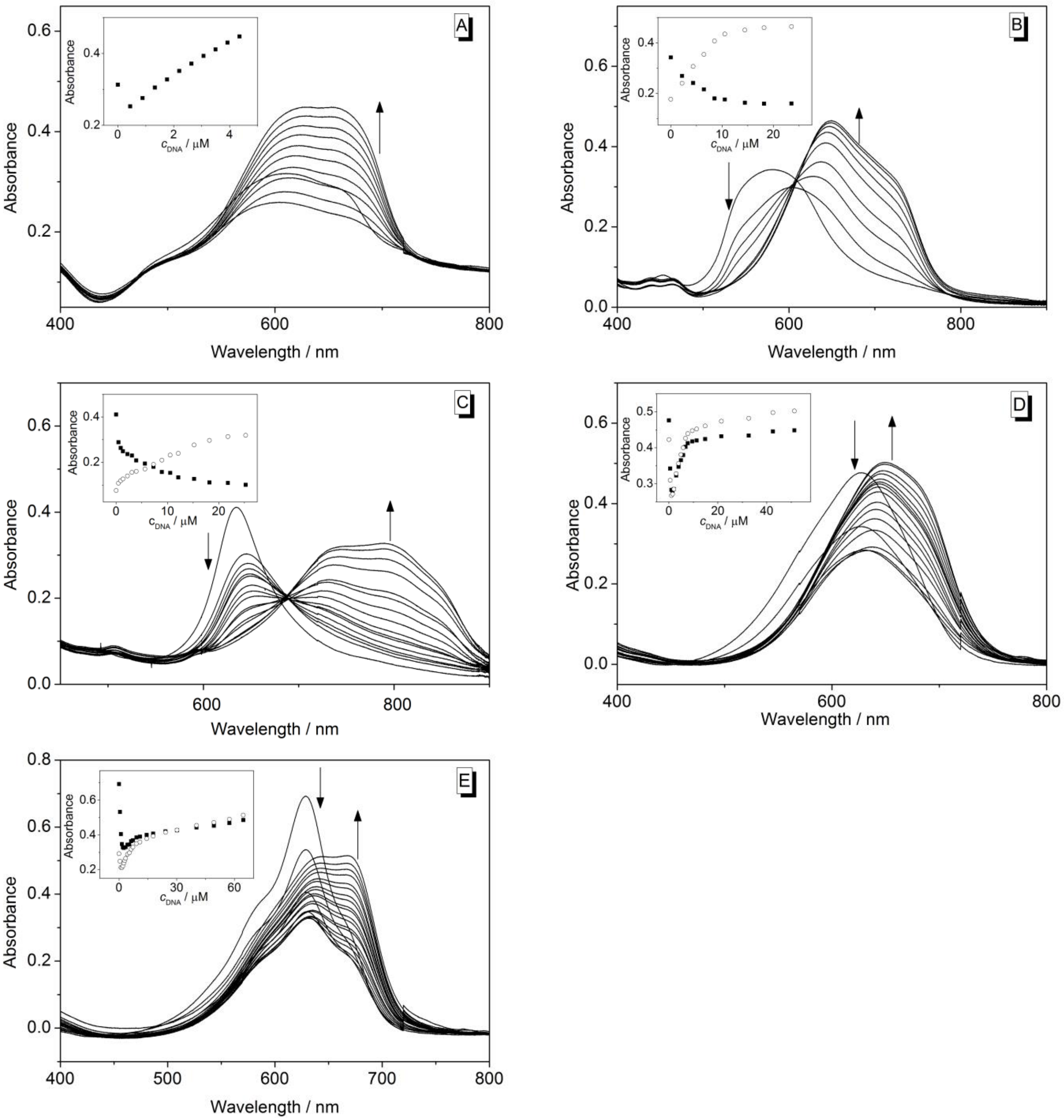

Figure S3: Photometric titration of **1a** (A), **1b** (B), **1c** (C), **1d** (D) and **1e** (E) with **a2** in potassium phosphate buffer (95 mM, pH 7.0); A, B: c_{Lig} = 50 μM; C: c_{Lig} = 5.0 μM; D, E: c_{Lig} = 10.0 μM. Arrows indicate the development of the bands with increasing DNA concentration. Inset: Plot of the development of absorption bands during titration versus DNA concentration.

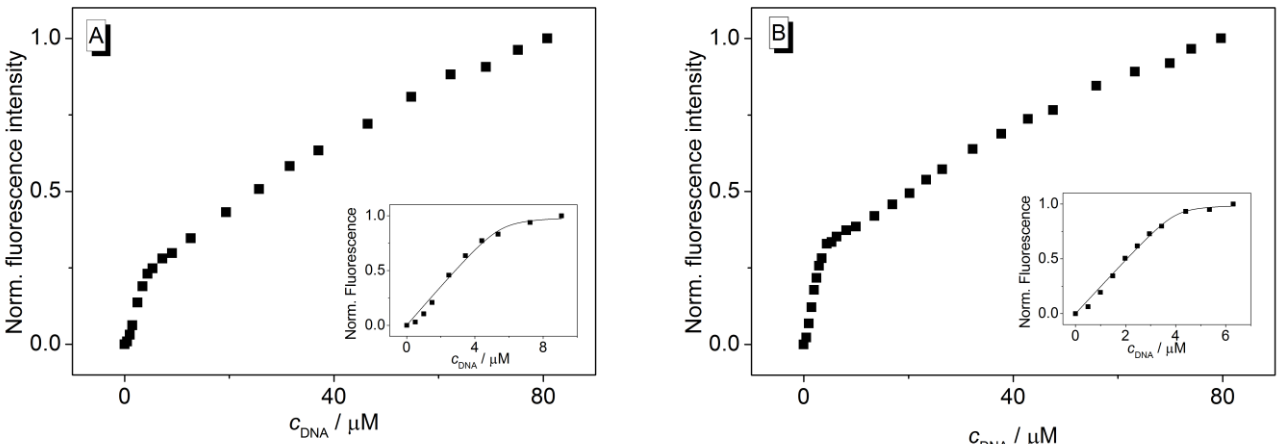

Figure S4: Plot of the change of the emission intensity of **1d** (A) and **1e** (B) versus DNA concentration. Inset: The fitting curve at at *LDR* = 1.1 (A) and 0.8 (B). A: c_{Lig} = 10 μM; B: c_{Lig} = 5 μM; λ_{ex} = 580 nm.

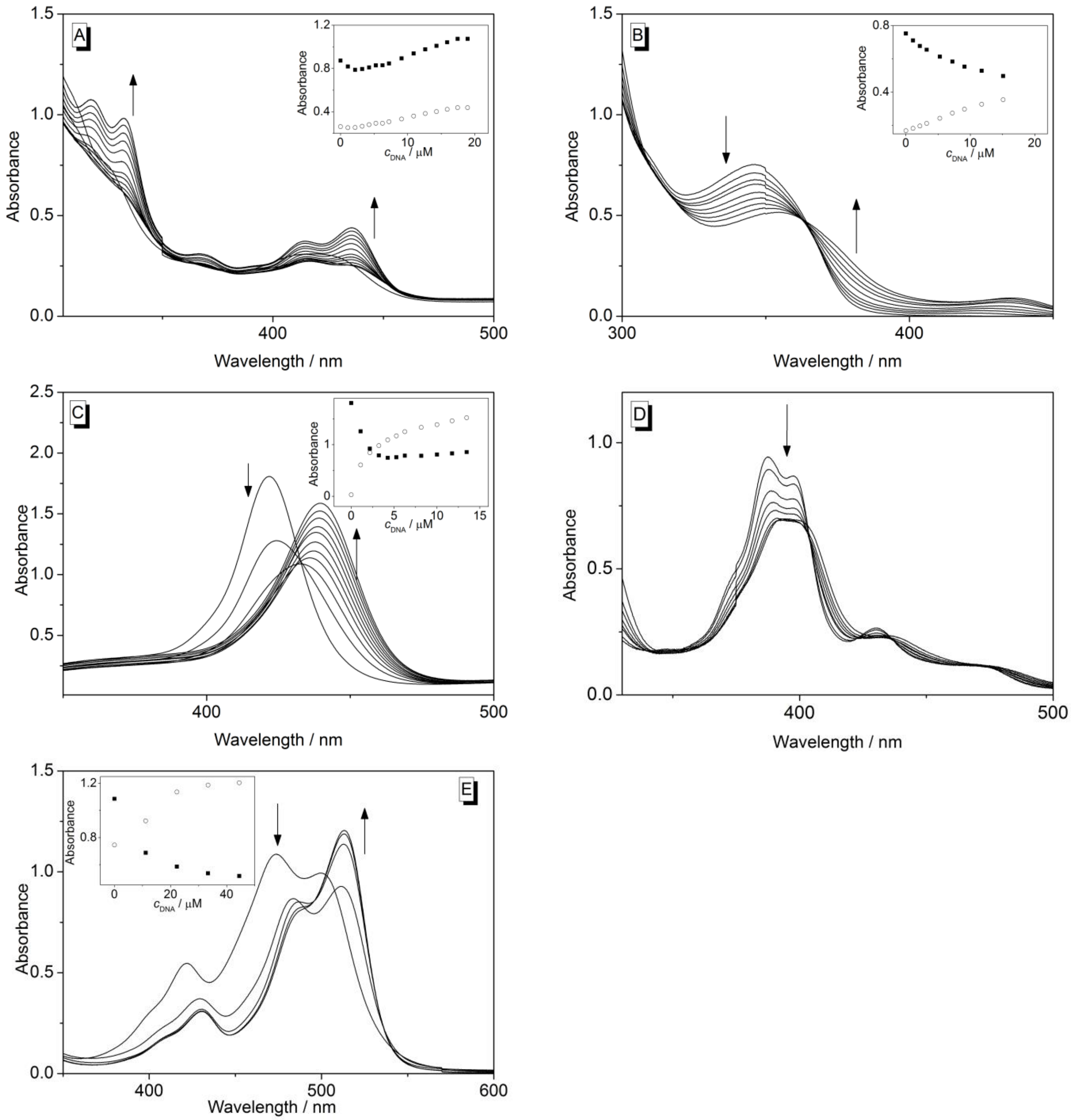

Figure S5: Photometric titration of **2** (A), **3** (B), **4** (C), **5** (D) and **6** (E) with **a2** in potassium phosphate buffer (95 mM, pH 7.0); A, B, D, E: c_{Lig} = 50 μM; C: c_{Lig} = 5.0 μM. Arrows indicate the development of the bands with increasing DNA concentration. Inset: Plot of the development of absorption bands during titration versus DNA concentration.

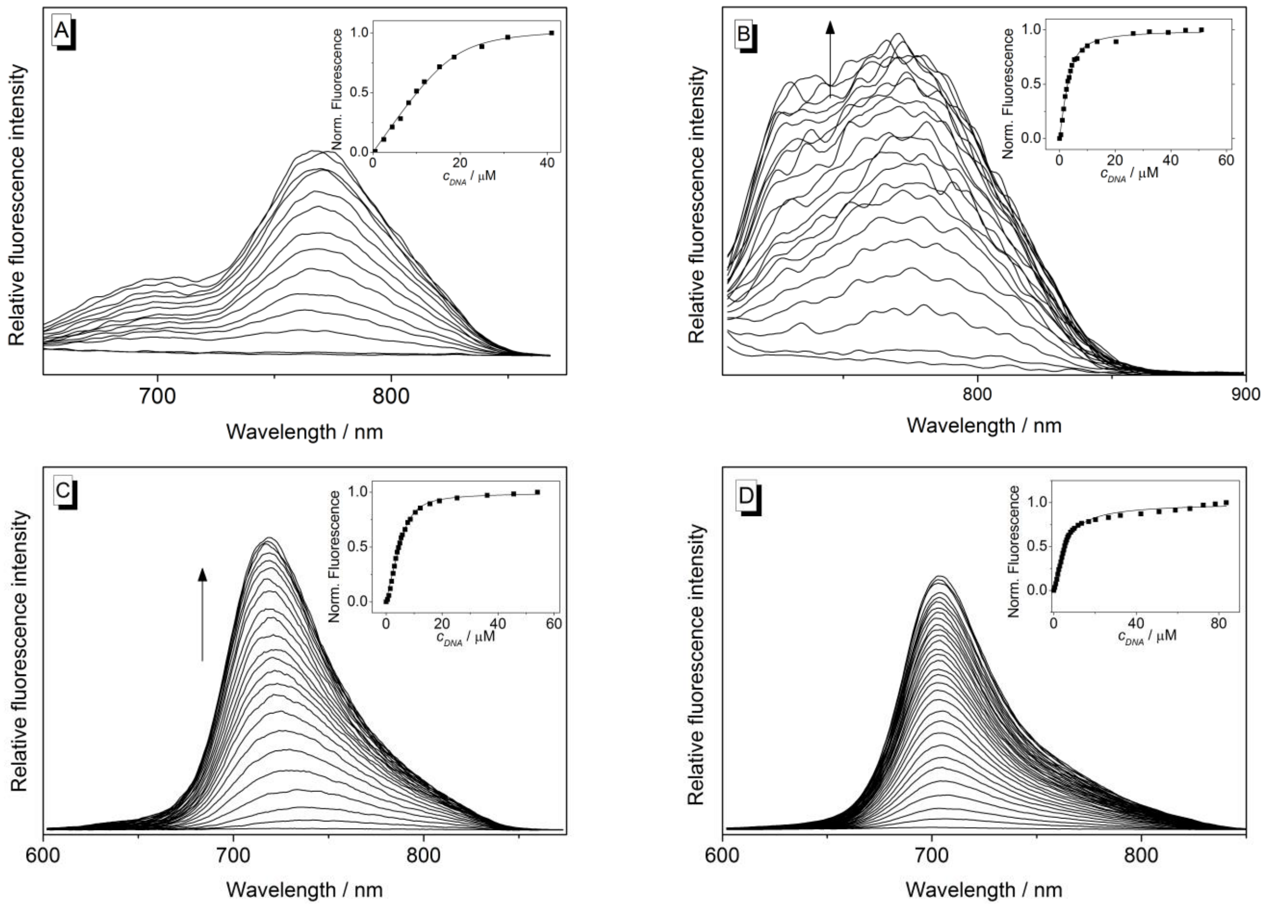

Figure S6: Fluorimetric titration of **1b** (A), **1c** (B), **1d** (C) and **1e** (D) with **22AG** in potassium phosphate buffer (95 mM, pH 7.0); A-C: c_{Lig} = 10.0 μM; D: c_{Lig} = 5.0 μM. Arrows indicate the development of the bands with increasing DNA concentration. Inset: Plot of the change of the emission intensity versus DNA concentration with the corresponding fitting curves.

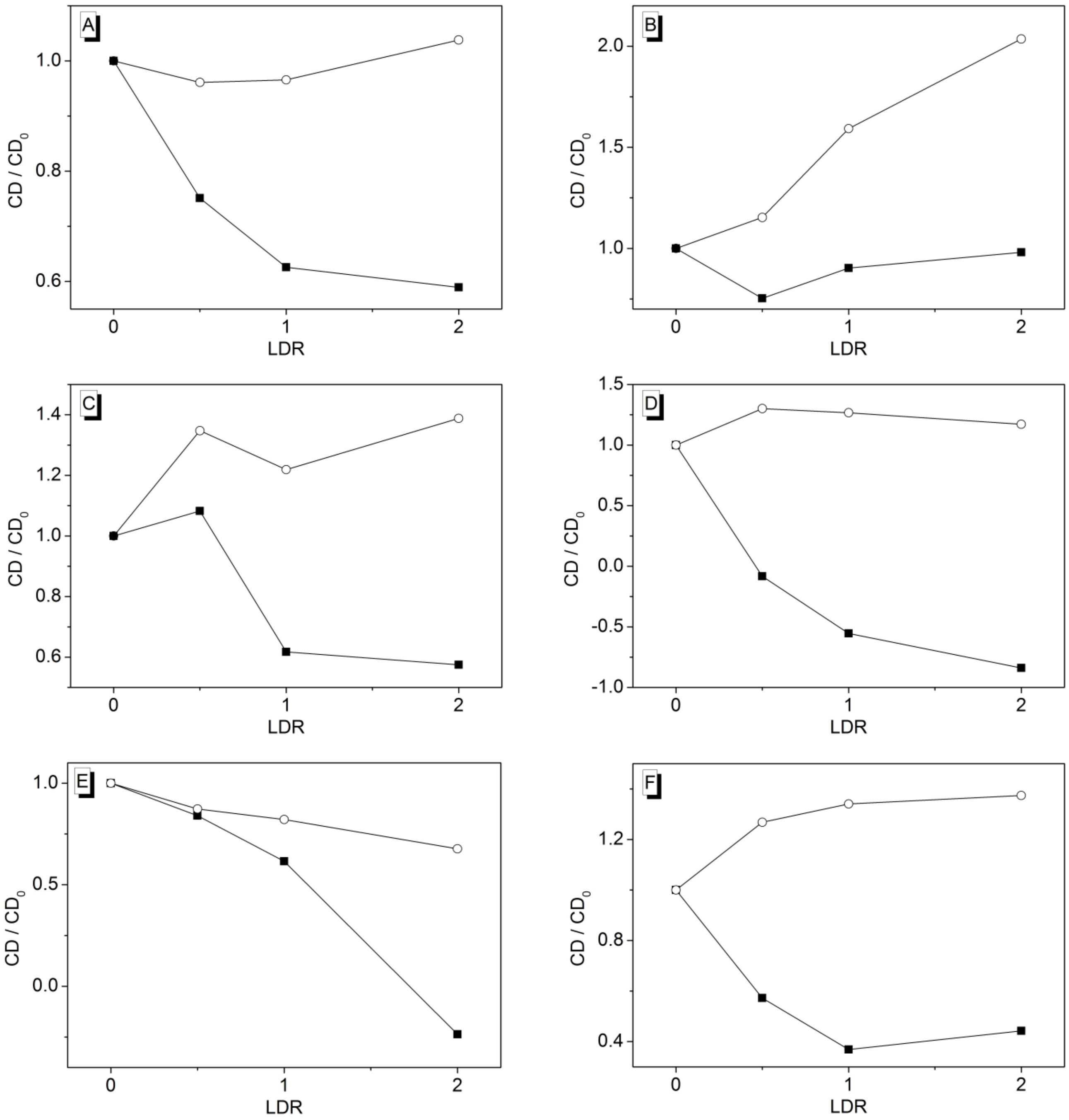

Figure S7: Plot of the intensity change (CD/CD_0) of the CD signals 265 nm (■) and 295 nm (○) of ILPR-DNA **a2** in the presence of **1d** (A), **1e** (B), **2** (C), **4** (D), **5** (E) and **6** (F) at *LDR* = 0, 0.5, 1, 2; c_{DNA}= 20 μM in potassium phosphate buffer (95 mM, pH 7.0); *T* = 20 ^{o}C.

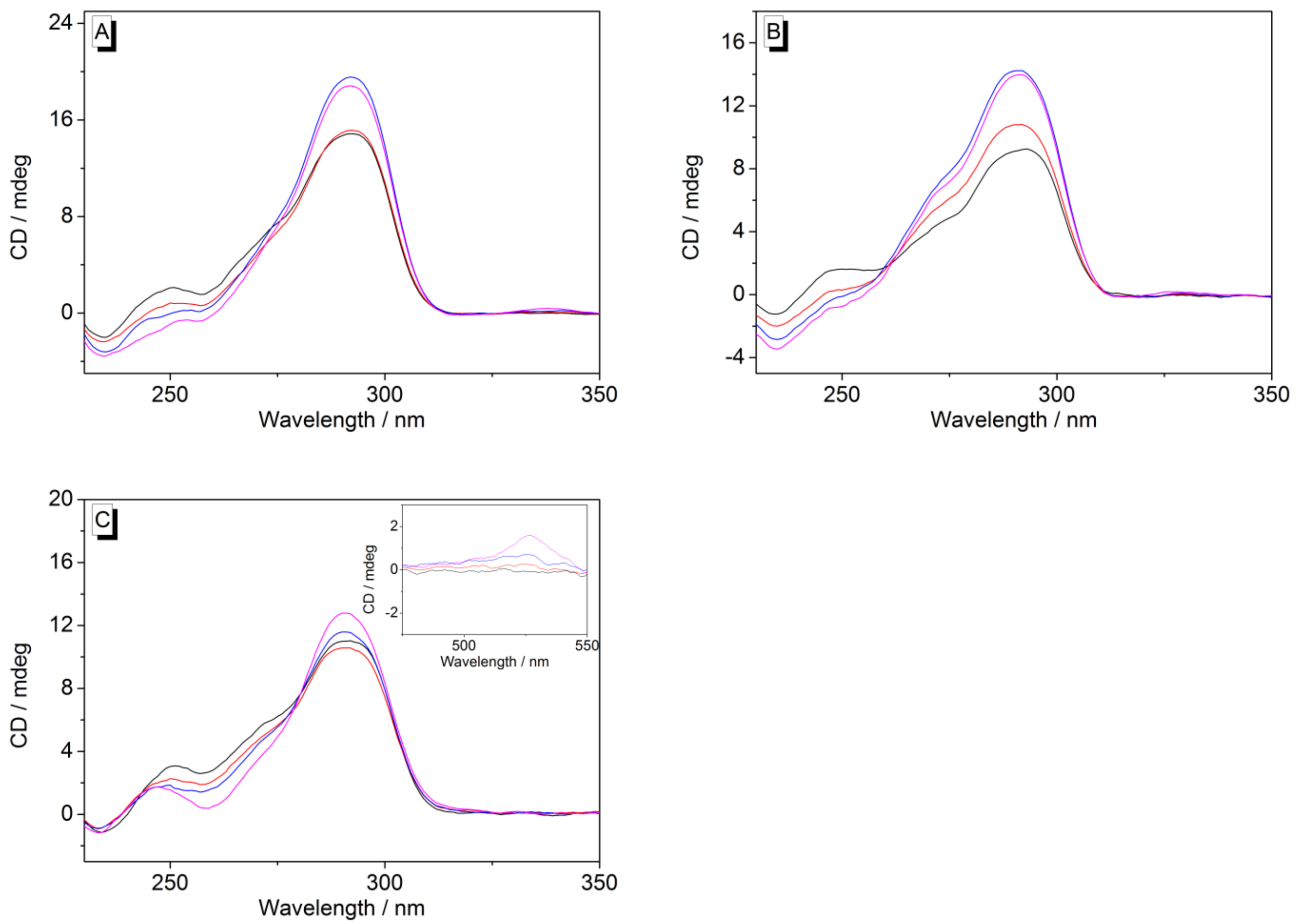

Figure S8: CD spectra of **22AG** in the presence of **1d** (A), **1e** (B) and **6** (C) at *LDR* = 0 (black), 0.5 (red), 1 (blue), 2 (magenta); c_{DNA} = 20 μM in potassium phosphate buffer (95 mM, pH 7.0); *T* = 20 °C. Inset: Magnified ICD signals of the bound ligands (magnification factor: ca. 10).
